# Effects of perinatal variables on echocardiographic assessments of left ventricular dimensions in infants born large for gestational age: a prospective cohort analysis

**DOI:** 10.1186/s13052-025-01945-5

**Published:** 2025-05-03

**Authors:** Ahmed Amarah, Ibrahim Elmakaty, Iram Nadroo, Manoj Chhabra, Danthanh Hoang, Debbie Suk, Ali M. Nadroo, Nitin Ron, Beata Dygulska, Madhu B. Gudavalli, Pramod Narula, Ashraf Gad

**Affiliations:** 1 College of Medicine, QU Health, Qatar University, Doha, Qatar; 2 Division of Neonatology, Department of Pediatrics, New York Presbyterian Brooklyn Methodist Hospital, 506 6 Th St, Brooklyn, NY 11215 USA; 3 Division of Critical Care, Neonatology, Women’S Wellness and Research Centre, Hamad Medical Corporation, Doha, Qatar

**Keywords:** Large for gestational age, Neonatal, Prospective cohort, Echocardiography, Linear regression, Left ventricular dimensions, Asymmetric septal hypertrophy, Perinatal factors

## Abstract

**Background:**

To assess the relationship between perinatal factors, and echocardiographic left ventricular (LV) dimensions after delivery in infants who are large for gestational age (LGA).

**Methods:**

This prospective cohort study that was conducted between 2014 and 2018, and involved healthy LGA newborns born ≥ 35 weeks’ gestation, delivered at New York-Presbyterian Brooklyn Methodist Hospital, and a control group of appropriate for gestational age (AGA) infants. Data were analyzed using multivariate linear regression in STATA.

**Results:**

A total of 563 neonates were enrolled in this study. They were composed of 414 AGA infants as the control group and 149 LGA infants as the intervention group. Males were predominant in both groups. A larger proportion of neonates were admitted to the neonatal intensive care unit (NICU) in LGA infants (74.6%) as compared to the AGA infants (33.5%) (*p* < 0.001). Regression analysis identified birth weight (BW) as a key factor, positively correlating with increased LVmass, interventricular septum thickness, and LV posterior wall thickness in both LGA and AGA infants. Additionally, BW showed a positive correlation with left ventricular internal dimensions in diastole and systole. Higher maternal BMI was associated with an increase in fractional shortening in LGA infants, while maternal insulin use during pregnancy was positively associated with interventricular septum thickness. Notably, male infants exhibited significantly higher LV internal dimensions in both diastole and systole, while GA negatively impacted the left ventricular mass index.

**Conclusion:**

The study's findings underscore the significant influence of perinatal factors on neonatal cardiac morphology in both LGA and AGA infants. Certain perinatal variables were identified as key determinants affecting various aspects of LV structure. These insights highlight the importance of considering these perinatal factors in neonatal cardiac assessments for early detection and intervention.

## Introduction

Large for gestational age (LGA) in newborns is defined as birth weight (BW) above the 90 th percentile for gestational age (GA), and it has been linked to a variety of maternal risk factors, including gestational diabetes mellitus (GDM). Cardiomyopathy is a common finding in LGA infants born to mothers with GDM and principally reflects maternal hyperglycemia during pregnancy [1]. The latter is considered a teratogenic state, leading to fetal complications, including adverse effects on cardiovascular development and fetal cardiac defects [2]. Although this relationship has been established, many LGA infants with cardiac changes, including thickening of the inter ventricular septum (IVS) and ventricular walls particularly of the left ventricle (LV), are born to mothers without a history of GDM, and this may be related to missed cases of GDM who failed to meet the definition criteria for GDM diagnosis, level of glycemic control during pregnancy or comorbidity such as high maternal body mass index (BMI) which is considered as an independent risk factor for perinatal complications [3].

Although fetal cardiac and vascular structural and functional changes are linked to maternal hyperglycemia [4], little is known about cardiac development and function in human children born to mothers with high BMI. In one study, 6-month-old neonates'LV mass increased in proportion to mother’s gestational weight growth [5]. Additionally, maternal hyperglycemia carries prenatal and perinatal risks and long-term risks for the mother and her child.

Cardiac remodeling and cardiovascular events in children are influenced by the shape and function of the LV. The LV geometry may stigmatize the morbidity and mortality in this population, even in asymptomatic conditions, such as before the start of overt hypertension or heart failure [6].

There is limited literature about the perinatal factors that affect the cardiovascular system of the newborn. Our recently published study showed that perinatal factors are significant predictors of LV parameters in small for GA babies [7]. Therefore, we conducted the current prospective cohort study which aimed to explore the relationship between perinatal factors, including maternal, and neonatal factors, and echocardiographic LV dimensions after delivery in LGA infants compared with babies who are appropriate for gestational age (AGA).

## Material and methods

### Study design and setting

This is a single-centered, prospective cohort study was used to assess the relation of perinatal factors with LV dimensions in newborn babies ≥ 35 weeks’ gestation. This study was carried out at New York-Presbyterian Brooklyn Methodist hospital in both neonatal intensive care (NICU) and nursery wards. This study was completed between 2014 and 2018.

### Study population

**Fig. 1:**
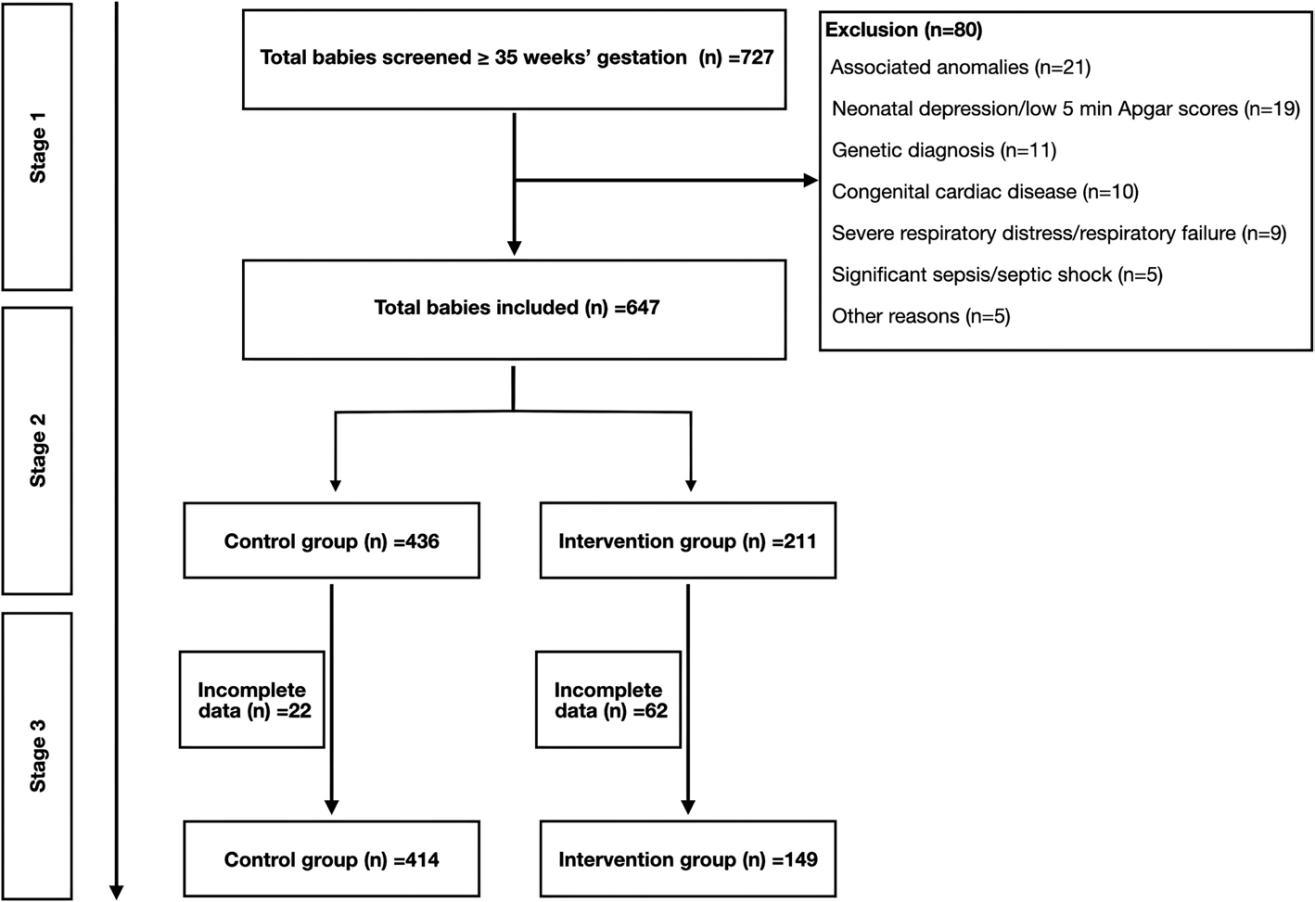
Recruitment process

The population in the current study consisted of newborns who were LGA. Echo evaluations were performed on all selected LGA babies as part of routine unit practice. The intervention and control groups were assigned based on Fenton growth charts published in 2003 and revised in 2013 [8]. The control group were all AGA babies who underwent echo studies for murmur evaluation before hospital discharge. Babies without congenital heart defects or major structural cardiac anomalies were included in the study. Figure 1 illustrates the exclusion criteria in details.

### Participants’ recruitment

At initial stage (stage 1) total 727 newborn babies were recruited, among them, 80 were excluded based on associated anomalies (*n* = 21), neonatal depression/low 5 min Apgar scores at 5 min (*n* = 19), genetic diagnosis (*n* = 11), congenital cardiac disease (*n* = 10), hypoxic respiratory failure (*n* = 9) and severe sepsis/shock (*n* = 5). A total of 647 participants were recruited after applying the inclusion criteria. The included participant was divided into two groups i.e., control group (AGA babies) and the intervention group (LGA babies). The baseline data were recorded at this stage (stage 2). At the final stage (stage 3) 22 participants were excluded from the control group and 62 were excluded from the intervention group because of missing data. The details are shown in Fig. 1.

### Ethical consideration

The current study was conducted as per the Declaration of Helsinki. The study was approved by the hospital institutional research board (IRB). No consent taken from their parents since all echostudies were routinely done on LGA infants in our NICU to rule out structural heart diseases. Confidentiality of the data was maintained by assigning a fictitious number to each participant and all the data was stored in a locked folder.

### Cardiac variables

Echo studeis were performed by using Philips 5500 Echocardiography machine (Philips, Andover, MA, USA) to evaluate the heart per unit protocol, only data on LV dimensions were collected including IVS thickness during systole and diastole (IVSs and IVSd, respectively), left ventricular internal dimension (LVID) during systole and diastole (LVIDs and LVIDd, respectively), left ventricular posterior wall (LVPW) thickness at end systole and diastole (LVPWs and LVPWd, respectively), IVS/LVPW ratio, shortening fraction (FS), left ventricular volumes (LV mass [LVmass] and LV mass index (g/m^2^).

### Data analysis

Microsoft Excel was used for data management (Microsoft Office, Redmond, Washington, United States) and statistical analysis was performed by using Stata version 16 (College Station, TX, USA). Asymmetric septal hypertrophy (ASH) was defined as the interventricular septum having a thickness greater than 6 mm and a ratio of septal to posterior wall thickness that is greater than 1.3 [9]. For the differences in baseline characteristics between LGA and AGA, we used two main tests: A chi-squared test was performed for the categorical variables while a simple two-way t-test was used for the continuous variables. Categorical data were presented as frequency and percentage while continuous data were presented as a mean and standard deviation. Any missing data was removed from the analysis tables. A logistic regression model was implied to assess the association of perinatal factors with LV dimensions. This model was performed over two steps. First, univariate linear regression was performed for all continuous variables. Then, multivariate linear regression was performed for significant variables. In the case of categorical variables such as ASH, a binary logistic regression was initially performed. Consequently, logistic regression was performed including only significant variables. The *P*-value was considered significant if < 0.05.

## Results

A total of 563 participants were included in the final analysis, among which 414 were in the control group (AGA babies) and 149 were in the intervention group (LGA babies). Table 1 demonstrates and compares the various neonatal and maternal variables between the LGA and AGA infants. Results showed that most of the participants in both groups were males (AGA = 51.45% and LGA = 61.7%). The mode of delivery was dominantly cesarean section (66.4%) in the LGA group, while in the AGA group, vaginal and c-section deliveries were equally distributed (50%). Among the AGA babies, 33.5% were admitted to NICU, while the LGA group, demonstrated higher rates of NICU admission (74.6%). The analysis of the Apgar scores at 1 and 5 min showed statistically insignificant results (*p* = 0.081 and *p* = 0.125 respectively). ASH was more prevalent among LGA infants (42.3%) as compared to AGA infants (28.0%), *p*=0.001. The table also demonstrates some descriptive biometric characteristics of the infants such as GA, BW, height, head circumference, chest circumference, and ponderal index (PI). On comparing the GA of LGA and AGA infants, it was found that it was almost similar in LGA infants (38.30 ± 1.20 WG) and AGA infants (38.69 ± 1.48 WG). The BW was expectedly significantly higher in LGA than AGA infants (4337.46 ± 404.45 g and 3381.05 ± 445.77 g respectively). Similarly, the other measurements such as height, head circumference, and chest circumference were significantly increased in LGA infants compared to AGA infants. The PI is another important factor that was measured. PI is the ratio of body weight to height and is calculated as (g/cm³) = [birth weight (g)] / [length (cm)]³ × 100t [10]. The mean PI in LGA infants was calculated as 3.11 ± 0.38 compared to the mean PI of AGA infants which was calculated as 2.79 ± 0.34.

**Table 1:** Neonatal and maternal variables in large for gestational age and appropriate for gestational age neonates

Variable	LGA		AGA		Total	*P*-value
	**f**	**%**	**f**	**%**		
**Neonatal Variables**						
**Mode of Delivery**						
Vaginal	50	33.56	204	49.64	254	0.001
Cesarean section	99	66.44	207	50.36	306	
**NICU Admission**						
No	29	25.44	147	66.52	176	< 0.001
Yes	85	74.56	74	33.48	159	
**Sex**						
Male	92	61.74	213	51.45	305	0.031
Female	57	38.26	201	48.55	258	
**Apgar (1 min)**						
Low	22	14.97	40	9.71	62	0.081
Normal	125	85.03	372	90.29	497	
**Apgar (5 min)**						
Low	4	2.72	4	0.97	8	0.125
Normal	143	97.28	408	99.03	551	
**Asymmetric Septal Hypertrophy**						
No	86	57.72	298	71.98	384	0.001
Yes	63	42.28	116	28.02	179	
**Gestational Age (weeks)**^**a**^	38.30 ± 1.20		38.69 ± 1.48			0.01
**Birth Weight (g)**^**a**^	4337.46 ± 404.45		3381.05 ± 445.77			< 0.001
**Birth Height (cm)**^**a**^	51.94 ± 2.24		49.50 ± 2.64			< 0.001
**Head Circumference (cm)**^**a**^	35.93 ± 1.40		34.31 ± 1.91			< 0.001
**Chest Circumference (cm)**^**a**^	36.26 ± 1.95		33.11 ± 2.01			< 0.001
**Ponderal Index (g/cm³)**^**a**^	3.11 ± 0.38		2.79 ± 0.34			< 0.001
**Maternal Variables**						
**Gravida**						
Primi	27	18.12	93	22.79	120	0.47
Multi	92	61.74	242	59.31	334	
Grand	30	20.13	73	17.89	103	
**Parity**						
Nulli	27	18.12	158	38.44	185	< 0.001
Multi	118	79.19	241	58.64	359	
Grand	4	2.68	12	2.92	16	
**Diabetes Mellitus**						
No	74	50.34	259	63.79	333	0.004
Yes	73	49.66	147	36.21	220	
**Preeclampsia**						
No	129	86.58	383	93.41	512	0.01
Yes	20	13.42	27	6.59	47	
**Ethnicity**						
White	52	40.94	179	51.59	231	< 0.001
Black	53	41.73	114	32.85	167	
Hispanic	14	11.02	8	2.31	22	
Asian	1	0.79	15	4.32	16	
Other	7	5.51	31	8.93	38	
**Maternal Age (years)**^**a**^	32.67 ± 5.63		31.55 ± 5.77			0.041
**Maternal BMI (kg/m²)**^**a**^	35.74 ± 7.49		31.68 ± 6.03			< 0.001

*Abbreviations*: *f *Frequency, *%* Percentage, *NICU* Neonatal Intensive Care Unit, *BMI* Body Mass Index, Low Apgar score if <7

^a^Values are Expressed as Frequencies and Percentages or Mean ± SD

As for the maternal variables, the AGA group were predominantly of white ethnicity (51.6%). However, in the LGA group, most infants were of black ethnicity (41.7%). Most of babies in the AGA group were born to non-diabetic mothers (63.8%) while in the LGA group there was almost an equal number of diabetic and non-diabetic mothers (50.3% vs 49.7%). Preeclampsia was not different in the mothers of both AGA and LGA infants (93.4% and 86.6% respectively). Maternal BMI was significantly higher in mothers of LGA infants (35.74 ± 7.49) compared to mothers of AGA infants (31.68 ± 6.03). Finally, gravidity yielded statistically insignificant results (*p* = 0.47). The details of the neonatal and maternal factors can be seen in Table 1.

Table 2 showed a detailed description of cardiac variables that were collected for all the infants in our study assessed by echo exams. It compares the means and standard deviation of those cardiac variables for LGA infants and AGA infants. All the variables were significant higher in the LGA group except the LVIDs (*p* = 0.19). The mean thickness of the IVS was significantly increased in LGA infants as compared to AGA infants (5.1 vs 4.0 mm in diastole, and 6.4 vs 5.3 mm in systole, respectively). The increased mean IVSs is due to the contraction of the muscle fibers that cause thickening of the IVS [11]. Some variables like LVIDd and LVPWd were minimally increased in LGA which might indicate clinical insignificance. The details of all the parameters can be found in the Table below.

**Table 2:** Echocardiographic cardiac parameters in large for gestational age and appropriate for gestational age neonates

Variable	LGA		AGA		N	*P*-value
	**Mean**	**SD**	**Mean**	**SD**		
LVmass (g)	14.12	4.00	10.27	3.26	559	< 0.001
LVmass index (g/m^2^)	58.89	14.80	49.49	12.15	549	< 0.001
LVIDd (mm)	19.41	2.27	18.57	2.11	559	< 0.001
LVIDs (mm)	12.14	1.84	11.93	1.62	559	0.19
IVSd (mm)	5.10	1.29	4.00	0.78	562	< 0.001
IVSs (mm)	6.40	1.37	5.34	1.05	559	< 0.001
LPWDd (mm)	4.05	0.73	3.48	0.61	559	< 0.001
LVPWs (mm)	5.48	0.87	4.83	0.70	558	< 0.001
IVS/LPW	1.28	0.39	1.17	0.28	557	< 0.001
FS (%)	37.49	5.20	35.56	4.51	559	< 0.001

*Abbreviations*: *IVS* Inter Ventricular Septum in Diastole (IVSd) and Systole (IVSs), *LVID *Left Ventricular Internal Dimension in Diastole (LVIDd) and Systole (LVIDs), *LVPW* Left Ventricular Posterior Wall in Diastole (LVPWd) and Systole (LVPWs), *FS* Fractional Shortening, *LV*mass *Index* *LV* Mass to Volume Ratio, *LGA *Large for Gestational Age, *AGA* Appropriate for Festational Age, *SD* Standard Deviation

Multivariate linear regression analysis revealed siginficant association between perinatal factors and LV echo parameters significant (Table 3). In the IVSd regression model, both GA and the Apgar score at 1 min had statistically significant negative effects on IVSd (R^2^ = 0.34, Adjusted [Adj] R^2^ = 0.34). Specifically, GA had a significant negative impact with a coefficient of − 0.140 (*p* < 0.001), and the Apgar score at 1 min also had a statistically significant negative effect, with a coefficient of − 0.066 (*p* = 0.004). However, Maternal insulin use during pregnancy and BW both had positive and significant effect on IVSd with a coefficient of 0.561 (*p* < 0.001) and 0.001 (*p* < 0.001) respectively. Similarly, BW was significantly associated positively with IVSs with a coefficient of 0.001 (*p* < 0.001),

**Table 3:** Multivariate regression analysis of perinatal factors associated with left ventricular parameters

LV parameter	N	Variable	Coeff	SE	*P*-value	R^2^, Adj R^2^
**IVSd (mm)**	547	GA	− 0.140	0.028	< 0.001	0.34, 0.34
		Birth weight	0.001	0.000	< 0.001	
		Apgar (1 min)	− 0.066	0.023	0.004	
		Insulin use	0.561	0.156	< 0.001	
*Other variables controlled for in this model: Maternal BMI						
**IVSs (mm)**	547	Birth weight	0.001	0.000	< 0.001	0.25, 0.24
*Other variables controlled for in this model: NICU admission, GA, Birth weight, Category, PI, Maternal BMI, Diabetes, Diabetic control, Preeclampsia						
**LVIDd (mm)**	547	Sex (female)	− 0.776	0.177	< 0.001	0.15, 0.14
		GA	0.149	0.067	0.027	
		Birth weight	0.001	0.000	< 0.001	
		Apgar (1 min)	0.100	0.054	0.062	
*Other variables controlled for in this model: Maternal BMI, Preeclampsia, Mean maternal BP						
**LVIDs (mm)**	555	Sex (female)	− 0.464	0.142	0.001	0.07, 0.06
		GA	0.133	0.054	0.014	
		Birth weight	0.000	0.000	0.007	
*Other variables controlled for in this model: Preeclampsia						
**LVPWd (mm)**	547	Birth weight	0.001	0.000	< 0.001	0.23, 0.23
*Other variables controlled for in this model: GA, Maternal BMI, Preeclampsia, Insulin use						
**LVPWs (mm)**	552	Birth weight	0.001	0.000	< 0.001	0.20, 0.20
*Other controlled for variables in this model: GA, Maternal BMI, Preeclampsia						
**FS (%)**	554	Birth weight	< 0.001	< 0.001	0.009	0.04, 0.04
		Maternal BMI	0.093	0.032	0.004	
*Other variables controlled for in this model: MOD						
**LVmass (g)**	552	Birth weight	0.004	0.000	< 0.001	0.33, 0.32
		Parity	0.223	0.119	0.063	
*Other variables controlled for in this model: Sex, GA, Maternal BMI, Preeclampsia						
**LVmass Index (g/m**^**2**^)	538	GA	− 0.920	0.410	0.029	0.13, 0.12
		Birth weight	0.008	0.001	< 0.001	
*Other variables controlled for in this model: Maternal BMI, Insulin use						

*Abbreviations*: *LVmass *Left Ventricular Mass, *LVmass Index* Left Ventricular Mass Index, *IVSd* Inter-Ventricular Septal thickness in Diastole, *IVSs* Inter-Ventricular Septal Thickness in Systole, *LVIDd* LV Internal Dimension in Diastole, *LVIDs* LV Internal Dimension in Systole, *LVPWd* LV Posterior Wall thickness in Diastole, *LVPWs* LV Posterior Wall Thickness in Systole, *IVS/LVPW* Inter-Ventricular Septal Thickness to LV Posterior Wall Thickness Ratio in Diastole, *FS* Shortening Fraction, *SD* Standard Deviation, *RMSE* Root Mean Square Error, *Coeff* Coefficient, *GA* Gestational Age, *BMI* Body Mass Index, *BP* Blood Pressure, *MOD* Mode of Delivery, *Adj *Adjusted

^*^Variables showing significant associations (*p *<0.1) in univariate analysis were included in the multivariate regression model

LVIDd regression results (*R*^2^ = 0.15, Adj *R*^2^ = 0.14) showed sex was found to be a significant predictor of LVIDd (*p* < 0.001), with a negative coefficient of − 0.776, indicating that male infants have a higher LVIDd than female infants. Similarly, in LVIDs regression model (*R*^2^ = 0.07, Adj *R*^2^ = 0.06), sex was found to be a significant predictor of LVIDs (*p* = 0.001), with a negative coefficient of − 0.464. GA was significantly associated with LVIDd (*p* = 0.027) and LVIDs (*p* = 0.014). Similarly, BW was associated with both LVIDd (*p* < 0.001) and LVIDs (*p* = 0.007). The regression results of LVPWd (*R*^2^ = 0.23, Adj *R*^2^ = 0.23) and the LVPWs (*R*^2^ = 0.20, Adj *R*^2^ = 0.20), BW was the only positive associated perinatal factor (*p* < 0.001). BW and maternal BMI were found to be positively associated with FS (*R*^2^ = 0.04, Adj *R*^2^ = 0.04). These results were statistically significant (*p* = 0.004 and *p* = 0.009 respectively), with a positive coefficient of < 0.001 and 0.093 respectively. This indicates that higher BW and maternal BMI are associated with an increase in FS.

The regression analysis for LVmass (R^2^ = 0.33, Adj R^2^ = 0.32) revealed that BW is a significant predictor of LVmass, with a positive coefficient of 0.004 (*p* < 0.001), suggesting that higher BW is associated with an increase in LVmass. In the case of the LVmass index regression model, the R^2^ value was 0.08, and the adjusted R^2^ value was 0.07. It showed a negative significant relationship between GA and LVmass index (*p* = 0.029). However, BW, was found to be a significant predictor with a positive association (*p* < 0.001).

Regarding the univariate binary regression, neither ASH nor IVS/LVPW showed any significant associations with the independent variables included in the analysis, and therefore, the results of this analysis were not included in the multivariate model.

The multivariate linear regression analysis performed to determine the association between perinatal factors and cardiac variables in both LGA and AGA infants are shown in Table 4. The data showed no significant association between any of the perinatal variables and LVmass in both LGA and AGA infants except for BW. There was a positive relationship between LVmass and BW as the coefficient was 0.003, meaning that for each unit (g) increase in BW the LVmass increases by 0.003g. This result was statistically significant (*p* < 0.001). Finally, there was also an association between preeclampsia and LVmass in the AGA infants (coeff – 1.222, *p* = 0.045). The same was not observed in the LGA group, as there was no statistical significance. Further details about the rest of the perinatal variables and their association with LVmass are presented in Table 3.

**Table 4:** Results of multivariate regression investigating the association between perinatal variables and cardiac parameters in appropriate for gestational age and large for gestational age infants

Cardiac Parameter	Appropriate for Gestational Age					Large for Gestational Age				
	**Observations (N)**	**Variable**	**Coefficient**	**SE**	***P*-value**	**Observations (N)**	**Variable**	**Coefficient**	**SE**	***P*-value**
LVmass (g)	405	Sex (female)	− 0.499	0.301	0.098	147	Sex (female)	− 0.119	0.658	0.857
		BW	0.003	< 0.001	< 0.001		BW	0.003	0.001	< 0.001
		Preeclampsia	1.222	0.608	0.045		Preeclampsia	0.326	0.986	0.742
*Other variables controlled for in these models: Parity, Maternal BMI										
LVmass index(g/m^2^)	398	BW	0.005	0.001	< 0.001	140	BW	0.006	0.003	0.060
*Other variables controlled for in these models: Maternal BMI, Insulin use										
IVSd (mm)	402	BW	< 0.001	< 0.001	< 0.001	145	BW	0.001	< 0.001	< 0.001
		Apgar1 (1 min)	− 0.095	0.024	< 0.001		Apgar (1 min)	− 0.002	0.053	0.965
		DM (Insulin)	0.399	0.171	0.020		DM (Insulin)	1.015	0.330	0.003
*Other variables controlled for in these models: Maternal BMI										
IVSs (mm)	401	BW	0.001	< 0.001	< 0.001	146	BW	0.001	< 0.001	< 0.001
		Preeclampsia	0.175	0.209	0.403		Preeclampsia	0.546	0.322	0.093
*Other variables controlled for in these models: Maternal BMI, DM, Insulin use										
LVIDd (mm)	402	Sex (female)	− 0.644	0.202	0.002	145	Sex (female)	− 0.833	0.369	0.025
		BW	0.002	< 0.001	< 0.001		BW	< 0.001	< 0.001	0.576
		Apgar (1 min)	0.161	0.066	0.014		Apgar (1 min)	− 0.044	0.092	0.638
		Preeclampsia	0.700	0.426	0.101		Preeclampsia	− 1.101	0.571	0.056
*Other variables controlled for in these models: Maternal BMI, Maternal mean BP										
LVIDs (mm)	407	Sex (female)	− 0.345	0.159	0.030	148	Sex (female)	− 0.635	0.308	0.041
		BW	0.001	< 0.001	< 0.001		BW	< 0.001	< 0.001	0.913
		Preeclampsia	0.170	0.319	0.596		Preeclampsia	− 1.146	0.456	0.013
*Other variables controlled for in these models: None										
LVPWd (mm)	402	BW	0.001	< 0.001	< 0.001	145	BW	< 0.001	< 0.001	0.003
		DM (Insulin)	0.257	0.137	0.061		DM (Insulin)	− 0.031	0.201	0.877
*Other variables controlled for in these models: Maternal BMI, Preeclampsia										
LVPWs(mm)	405	BW	0.001	< 0.001	< 0.001	147	BW	< 0.001	< 0.001	0.011
*Other variables controlled for in these models: Maternal BMI, Preeclampsia										
FS (%)	406	BW	< 0.001	0.001	0.423	148	BW	< 0.001	0.001	0.968
		Maternal BMI	0.065	0.038	0.086		Maternal BMI	0.135	0.059	0.025
*Other variables controlled for in these models: MOD										
IVS/LVPW		DM (Insulin)	0.025	0.066	0.703	145	DM (Insulin)	0.236	0.107	0.030
		Preeclampsia	0.078	0.059	0.185		Preeclampsia	0.178	0.096	0.066
*Other variables controlled for in these models: Birth weight										

*Abbreviations*: *LVmass* Left Ventricular Mass, *LVmass Index* Left Ventricular Mass Index, *IVSd* Inter-Ventricular Septal Thickness During Diastole, *IVSs* Inter-Ventricular Septal Thickness During Systole, *LVIDd* Left Ventricular Internal Dimension During Diastole, *LVIDs* Left Ventricular Internal Dimension During Systole, *LVPWd* Left Ventricular Posterior Wall Thickness During Diastole, *LVPWs* Left Ventricular Posterior Wall Thickness During Systole, *FS* Shortening Fraction, *SE* Standard Error, *GA* Gestational Age, *BW* Birth Weight, *BMI* Body Mass Index, *DM* Diabetes Mellitus, *DM (insulin)* Diabetes Mellitus Controlled by Insulin Medication, *MeanBP* Mean Blood Pressure, *MOD* Mode of Delivery

Similarly, there was a positive association between BW in the AGA infants and LVmass index with a coefficient of 0.005 (*p* < 0.001). However, there was no statistical significance observed in the LGA group (*p* = 0.06). The remaining perinatal factors (maternal BMI and insulin-controlled DM) had no statistical significance in either group. Analysis showed that the IVS was significantly affected by perinatal factors. However, there were some discrepancies between their effects on the IVS during diastole and systole. For instance, during diastole the IVS thickness had a statistically significant positive association with perinatal factors such as BW (*p* < 0.001), and insulin-controlled DM (*p* = 0.02) in the AGA group. It also had a statistically significant negative association with Apgar score at 1 min (*p* < 0.001) in the same group. In the LGA group, there was a positive association between IVS thickness during diastole and BW (*p* < 0.001), and insulin-controlled DM (*p* = 0.003). On the contrary, the thickness of IVS during systole had a statistically significant positive association with only BW (*p* < 0.001) in both groups.

The association between perinatal factors and both LVIDd and LVIDs were also analyzed. Sex was the most notable finding as the results showed a strong association between male gender and having an increased LVIDd in AGA and LGA infants (*p* = 0.002 and *p* = 0.025 respectively). Similarly, there was a strong association between being a male and having an increased LVIDs in AGA and LGA infants (*p* = 0.03 and *p* = 0.041). BW was also found to be statistically correlated with LVIDd and LVIDs only in the AGA group (*p* < 0.001). Apgar score at 1 min had a positive association with LVIDd in AGA infants (coeff = 0.161 and *p* = 0.014). Finally, preeclampsia had a negative correlation with LVIDs in LGA infants (coeff = − 1.146 and *p* = 0.013).

Consistently, BW was positively correlated with LVPW during diastole in AGA and LGA infants (*p* < 0.001 and *p* = 0.003). Similarly, BW was positively correlated with LVPW during systole in both groups (*p* < 0.001 and *p* = 0.011, respectively). In addition, there was a statistically significant positive association between maternal BMI and FS in the LGA infants (*p* = 0.025). FS was not found to be positively correlating with any other perinatal factors.

The final variable that was analyzed is the IVS/LVPW ratio. The cutoff for this value is used to determine ASH. The analysis revealed insulin-controlled DM as the only positively correlating variable with IVS/LVPW in LGA infants (coeff = 0.236, *p* = 0.03). The rest of the variables were statistically insignificant. Details are listed in Table 4. 

Significant perinatal variables (*p *<0.1) were included in the multivariate model to identify independent predictors of ASH (Table 5). Only decreasing GA and increasing BW were significantly associated with ASH, with adjusted odds ratios (aOR) of 0.785 (*p* = 0.001) and 1.005 (*p* = 0.001), respectively. It was also observed that the odds of developing ASH in LGA infants compared to AGA infants (OR = 1.372; 95% confidence interval: 1.291 – 1.667; *p* = 0.001).

**Table 5:** Results of multivariate binary logistic regression analyzing the association between asymmetric septal hypertrophy and perinatal factors in all infants

Asymmetric Septal Hypertrophy				
Observations (N= 553)				
**Variable**	**Adjusted Odds Ratio**	**SE**	**z-value**	***P*-value**
Gestational Age (weeks)	0.785	0.055	− 3.40	0.001
Birth Weight (g)	1.005	0.001	3.30	0.001
Apgar (1min)	0.901	0.050	− 1.87	0.062
Maternal mean BP (mmHg)	1.015	0.009	1.71	0.087
Intercept	446.41	1267.73	2.15	0.032

*Abbreviations*: *SE* Standard Error, *BP *Blood Pressure

## Discussion

The current study showed that most of the sampled infants that were admitted to the NICU were LGA. Similarly, ASH was found to be more prevalent in the LGA group as compared to AGA infants. In addition to the role of genetic and hereditary conditions, the study findings indicate that several perinatal variables play an important role in shaping cardiac morphology particularly LV structure. Our binary logistic regression demonstrated that GA had a significant but negative association with ASH, contributing more strongly than more than the positive association observed with BW, particularly in LGA infants. We hypothesize that this may be explained by interrupted cardiac remodeling or immature myocardial adaptation in response to intrauterine stressors such as hyperinsulinemia or hypoxia, particularly in LGA fetuses with lower GA. Moreover, LGA infants are often delivered earlier due to maternal conditions such as GDM, preeclampsia, or macrosomia, which may further contribute to altered cardiac development. Hyperglycemia in mothers with GDM is a teratogenic condition that affects organogenesis and induce epigenetic changes primarily through insulin production, modifications Melanocortin 4 receptor, lipoprotein lipase, and leptin methylation patterns, and increased adiposity in the growing fetus [12–15]. This fetal programming has a crucial role especially during the first 1000 days after conception leading to transgenerational consequences, including higher incidences of metabolic syndrome, overweight and cardiovascular abnormalities in older children and adult offspring of affected mothers [14–18].

ASH can develop in newborns of diabetic mothers; however, this association is not consistently supported in the literature, and the relationship between ASH and maternal diabetes remains controversial. While some studies suggest an increased odds of ASH in infants of diabetic mothers, our results found no significant association between ASH and LGA infants born to diabetic mothers [9, 19, 20]. Further studies are needed to better understand how variations in GA and BW influence patterns of cardiac remodeling and myocardial maturation in fetuses and neonates. The findings of the current study were consistent with the reported study from China where it was reported that children born to diabetic mothers and mothers with high BMIs showed persistently increased IVS thickness and decreased FS indicating ventricular dysfunction [21].

A significant association was found between BW and cardiovascular health, including LV mass, LV mass index, IVSD, IVSS, LVID, LVID, LVPD, and LVPD. The positive and highly significant association between BW and LV mass in both AGA and LGA infants is in line with previous studies. Based on a study by Sawyer et al., LV mass index was positively associated with birth BMI [22]. A larger LV mass might be an indication of better cardiac development during fetal growth or a potential adaptation to intrauterine conditions for infants with higher BWs. Further supporting the idea that BW plays a crucial role in influencing cardiac parameters is the positive association between BW and IVSd in both groups. However, another study by Vijayakumar et al. that examined the relationship between infant growth and LV mass in adulthood found no significant association between LV mass and BW [23]. The relationship between weight at one year and LV mass was independent of factors in adult life such as body size, systolic blood pressure, and age. The enlarged LV mass associated with reduced growth in infancy was concentric, affecting both the IVS and the LVPW. Therefore, further studies are needed to explore this relationship.

It has been previously reported that the weight of the child at birth is the significant predictor of BMI at a later age. A cohort study reported from Australia found that higher BW was a significant predictor of increased BMI in later age [24]. The higher BMI is a key contributing factor to the obesity pandemic observed in this poulation. Subsequently, obesity has a strong link with cardiovascular disease [25, 26]. From here we postulate that an increased BW can pose future cardiovascular health issues at a later stage in life that may not be evident at birth. We believe our study could provide base to future research that study the relation between BW and future cardiac issues.

Maternal obesity, gestational hypertension, and diabetes have been shown to significantly impact the structure and function of the LV, often leading to cardiac abnormalities. The ongoing obesity pandemic affects women of childbearing age and increases the risk of cardiovascular disease and cardiomyopathies[27]. Pregnancy causes metabolic changes such as increases in body weight, circulation lipids, glucose, and inflammatory markers. Obese women experience more of these changes than normal-weight women[6]. The uterine environment influences fetal organ development, influencing disease susceptibility throughout childhood, adolescence, and old age. According to epidemiological research, maternal obesity increases the risk of cardiovascular disease and premature mortality in adult and elderly children [28]. Heart development occurs mostly throughout childhood, although little is known about cardiac development and function in human children born to obese mothers. In one research, 6-month-old neonates'LV mass increased in proportion to mother gestational weight growth [5]. GDM carries prenatal and perinatal risks as well as long-term risks for the mother and her child. Fetal cardiac and vascular structural and functional changes are linked to maternal hyperglycemia [4]. Congenital cardiac abnormalities and hypertrophy in the offspring of Type1/Type2 DM mothers are widely documented. It has been reported that fetal hyperinsulinism is caused by intrauterine exposure to high maternal blood glucose and affects the liver and cardiovascular system the most structurally and functionally [29, 30].

Furthermore, the study showed an association between insulin treatment in diabetic mothers and cardiac parameters such as IVSd (coeff = 1.015) and IVS/LVPW (coeff = 0.236) in LGA infants. A study that investigated the effect of diabetic maternal insulin intake during pregnancy on fetal cardiac parameters yielded similar results [31]. The study demonstrated that fetuses of diabetic mothers had a higher mean cardiac output than their non-diabetic counterparts at 26–28 weeks of gestation (192.9 ± 67.74 vs 130.9 ± 20.3 respectively). Similarly, they also had an increased mean myocardial performance index (0.583 ± 0.06 vs 0.493 ± 0.06). Additionally, the same study reported an increase in mean cardiac output (316.057 ± 92.82 vs 251.188 ± 75.88) and mean myocardial performance index (0.62 ± 0.07 vs 0.58 ± 0.07) at 34–36 weeks gestation [31].

In addition, mothers who were diagnosed with preeclampsia had a significant positive association with LVmass in the AGA group. However, preeclampsia did not significantly correlate with LVmass in LGA newborns. This suggests that the effect of this condition on cardiac health may differ depending on the fetal and cardiac growth pattern. A study that investigated the effects of preeclampsia on neonatal cardiac development concluded that the LVmass index was unchanged at birth in infants of hypertensive mothers during pregnancy. However, at 3 months of age, those infants had significantly greater LVmass index [32].

These findings align with previous research conducted in the same population, which also demonstrated the influence of perinatal factors such as BW, GA, maternal BMI, and weight to placental weight ratio on LV size and structure [33, 34]. This underscores the critical role of fetal growth and placental function in the normal physiological development of the fetal heart. Cardiac remodeling and cardiovascular events in children are influenced by the shape and function of the LV. The LV geometry may stigmatize the morbidity and mortality in this population even in asymptomatic conditions, such as before the start of overt hypertension or heart failure [6]. We believe that further research is needed to study the long-term implications of these cardiac variables in LGA infants. Such studies could provide valuable insights and enhance our understanding of this field.

## Study limitations

The current study is subjected to various limitations that may affect its internal and external validity. Firstly, this was a single-centered cohort study, therefore this study cannot be generalized to all LGA babies across the population. Secondly, the small sample size may have negatively affected the power of the study in return affecting its ability to yield more significant associations. In addition, the number of participants in the intervention group was low as compared to the control group which may have affected the external validity of the study. Moreover, during the follow-up, the number of missed participants was high, and this may affect the outcome as it was dependent on the baseline data. Finally, this study had no follow-up of neonates at a later stage in their life. This limits the study’s ability to study the long-term outcomes of the evaluated maternal and neonatal variables on the cardiac parameters.

## Conclusion

The study's findings highlight the significant influence of perinatal factors on neonatal cardiac morphology in both LGA and AGA infants. BW, maternal BMI, and maternal insulin use during pregnancy were key determinants affecting various aspects of LV structure, including mass, wall thickness, and internal dimensions. GA negatively impacted the LV mass index, highlighting its influence on cardiac structural development. Additionally, gender differences in cardiac dimensions were evident, with male infants displaying larger LV dimensions. These insights highlight the importance of considering these perinatal factors in the assessment and monitoring of neonatal cardiac health, offering valuable guidance for tailored clinical approaches in pediatric cardiology. These findings underscore the complex interplay of perinatal factors in influencing neonatal cardiac structure, critical for pediatric cardiac evaluations. However, we believe that due to the limitations of our study, the data cannot be extrapolated to the global population. Further research is needed to explore the association between perinatal factors and cardiovascular health in infants and older children.

## Data Availability

The data used for the analysis in this work are available upon reasonable request from the corresponding author.
